# Identification of the HSP70-II gene in *Leishmania braziliensis *HSP70 locus: genomic organization and UTRs characterization

**DOI:** 10.1186/1756-3305-4-166

**Published:** 2011-08-26

**Authors:** César A Ramírez, José M Requena, Concepción J Puerta

**Affiliations:** 1 Laboratorio de Parasitología Molecular, Departamento de Microbiología, Facultad de Ciencias, Pontificia Universidad Javeriana, Carrera 7 No. 43-82, Edificio 52, Oficina 608, Bogotá, Colombia; 2 Centro de Biología Molecular "Severo Ochoa" (CSIC-UAM), Universidad Autónoma de Madrid, 28049 Madrid, Spain

## Abstract

**Background:**

The heat stress suffered by *Leishmania sp *during its digenetic life-cycle is a key trigger for its stage differentiation. In *Leishmania *subgenera two classes of *HSP70 *genes differing in their 3' UTR were described. Although the presence of *HSP70*-*I *genes was previously suggested in *Leishmania (Viannia) braziliensis*, *HSP70*-*II *genes had been reluctant to be uncovered.

**Results:**

Here, we report the existence of two types of *HSP70 *genes in *L. braziliensis *and the genomic organization of the *HSP70 *locus. RT-PCR experiments were used to map the untranslated regions (UTR) of both types of genes. The 3' UTR-II has a low sequence identity (55-57%) when compared with this region in other *Leishmania *species. In contrast, the 5' UTR, common to both types of genes, and the 3' UTR-I were found to be highly conserved among all *Leishmania *species (77-81%). Southern blot assays suggested that *L. braziliensis HSP70 *gene cluster may contain around 6 tandemly-repeated *HSP70*-*I *genes followed by one *HSP70*-*II *gene, located at chromosome 28. Northern blot analysis indicated that levels of both types of mRNAs are not affected by heat shock.

**Conclusions:**

This study has led to establishing the composition and structure of the HSP70 locus of *L. braziliensis*, complementing the information available in the GeneDB genome database for this species. *L. braziliensis HSP70 *gene regulation does not seem to operate by mRNA stabilization as occurs in other *Leishmania *species.

## Background

The *Leishmania *genus involve*s *about 20 species that infect humans, causing different clinical manifestations ranging from self-healing cutaneous lesions (CL), mucosal lesions (MCL) to fatal visceral infections (VL) [1]. More than 350 million people are considered at risk of contracting leishmaniases, and some 2 million new cases occur yearly [2]. In Latin America, CL and MCL are neglected public health problems endemic in 22 countries. Many species of the subgenus *Viannia *cause the majority of CL cases but MCL is principally caused by *Leishmania (Viannia) braziliensis *[3]. Canine leishmaniases, caused either by *Leishmania infantum *or by *L. braziliensis*, is also widespread in South America, being among the most important canine vector-borne diseases occurring in this region [4].

During its digenetic life cycle, the *Leishmania *parasite needs to adapt from the environmental (vector) temperature to the mammalian-host temperature (37°C). As a result, the heat shock response is induced and the heat shock proteins (HSPs) are expected to play important roles in the adaptation process, influencing the developmental change from promastigotes in sandflies to amastigotes in mammalian hosts [5-10]. Among HSPs, HSP70 is the most highly conserved in both sequence and function. Proteins of the HSP70 family are central components of many fundamental cellular processes, including the folding and assembly of newly synthesized proteins, refolding of misfolded and aggregated proteins, membrane translocation of organellar and secretory proteins, proteolytic degradation of unstable proteins, and control of regulatory protein activity [11-14].

Two classes of *HSP70 *genes, *HSP70-I *and *HSP70-II*, sharing the 5' untranslated region (UTR) and the coding region but differing in their 3' UTR, have been described in several *Leishmania *species like *L. infantum, Leishmania major, Leishmania tropica, Leishmania mexicana *and *Leishmania amazonensis *[15,16]. In general, these genes are arranged in a single genomic cluster that contains five or six *HSP70*-*I *copies, followed by one *HSP70*-*II *copy. In *L. infantum, *it has been demonstrated that whereas *HSP70*-*I *mRNAs accumulate in response to heat shock treatment, and are translated at both 26 and 37°C, *HSP70*-*II *mRNAs do not show a temperature-dependent accumulation, but show preferential translation at heat shock temperatures [15].

Given that *Leishmania *genes are transcribed into polycistronic RNA precursors that need to be further processed into individual mRNAs by *trans-*splicing and polyadenylation, post-transcriptional regulation represents the main level of controlling gene expression in these parasites [17]. Currently, multiple efforts are being dedicated to identify *cis- *and *trans-*elements involved in the modulation of mRNA processing, mRNA stabilization/destabilization, mRNA half-life, or translation efficiency. Although regulatory sequences have been identified in both 5' and 3' UTRs, most of them have been located in the 3' UTRs [18-23]. For instance, preferential translation of HSP83 in *Leishmania *requires a thermosensitive polypyrimidine-rich element (PPT) in the 3' UTR [24].

Our knowledge on the organization and expression of *HSP70 *genes in *Leishmania *species of the subgenus *Viannia *is scanty. Although, the sequence for the *L. braziliensis *genome has recently been published [25], unfortunately, the genomic sequence found in the GeneDB database presents several gaps that hinder to elucidate the organization of the *HSP70 *locus in *L. braziliensis*. Moreover, in a preliminary work, it was documented by hybridization assays the existence of *HSP70-I *genes in *L. braziliensis*, but evidence on the presence of *HSP70-II *genes was not obtained [16]. In this work, we have determined the 5' and 3' UTRs for the *HSP70 L. braziliensis *genes, demonstrating the existence of the *HSP70-II *gene in this *Viannia *species, and established the organization of the *HSP70 *locus. Also the expression of both types of *HSP70 *genes was assessed.

## Methods

### Parasite cultures

Promastigotes of *L. braziliensis (*MHOM/BR/75/M2904) were cultured *in vitro *at 26°C in Schneiders`s insect medium (Sigma Aldrich, Inc., St. Louis, USA) supplemented with 20% heat-inactivated fetal calf serum (Eurobio, Inc., Les Ulis, France), and 0.1 μg/mL of 6-Biopterin (Sigma Aldrich, Inc., St. Louis, USA). For heat shock treatments, 10 mL-aliquots of *L*. *braziliensis *promastigote cultures at logarithmic phase (5-9 × 10^6 ^promastigotes mL^-1^) were transferred into 50 mL flasks, and incubated at 32°C, 35°C or 37°C for two hours. Afterwards, parasites were harvested for DNA or RNA extraction.

### Southern and Northern blot analyses

Total DNA from *L. braziliensis *cells was isolated using the phenol-chloroform-isoamilic alcohol method [26]. Two μg of DNA were digested with several restriction enzymes according to the manufacturer specifications (Promega, Inc., Madison, WI, USA), electrophoresed on 0.8% low electroendosmosis agarose gels (Conda Pronadisa, Inc., Madrid, Spain), and transferred to nylon membrane (Roche, Inc., Mannheim Germany) by standard methods [26]. Total RNA from promastigotes was isolated using the Total Quick RNA Cells and Tissues (TALENT, Inc., Trieste, Italy). Four μg per line were separated on 1.5% (w/v) low electroendosmosis agarose/MOPS/formaldehyde gels and transferred to nylon membranes. *L. braziliensis *chromosomes were prepared from promastigotes, harvested during log phase, washed and embedded in 0.6% low melting agarose (GIBCO BRL, Inc., N.Y, USA) plugs, which were finally soaked in a lysis solution (0.5 M EDTA, pH 9; 1% SDS, 1 mg/mL proteinase K) at 50°C during 48 h.

After three washes with 0.2 M EDTA for 2 h each one, the blocks were loaded directly into the wells of 1% agarose NA gel (Amersham Bioscience, Inc., Uppsala, Sweden), sealed in place, separated by pulsed homogeneous electric field gel electrophoresis (PFGE) using a Pharmacia Biotech Gene Navigator apparatus at 100 V, 120° separation angle and switch times varying from 100 s/7 h; 200 s/10 h and 500 s/20 h, and transferred to nylon membranes. The following probes were used: 3' UTR-I (clone pTLb3HSP70-D, *Sma*I/*Eco*RI digested), 3' UTR-II (clone pTLb3H70-11B, *Hin*cII/*Eco*RI digested), and intercoding (IR-HSP70, clone pLbHSP70-IR-E, *Pst*I digested). They were labeled with digoxigenin by randomly primed synthesis using the DIG High Prime DNA Labeling kit (Roche, Inc., Mannheim, Germany). Hybridizations and immunological detection were performed using the Detection Starter kit II (Roche, Inc., Mannheim, Germany) according to manufacturer's instructions. Finally, membranes were exposed on Curix RP2 plus medical X-Ray film (AGFA, Mortsel, Belgium).

### Cloning UTR sequences and *in silico *analyses

First-strand cDNA synthesis was carried out from *L. braziliensis *total RNA using an oligo-dT primer and a cDNA synthesis kit (LKB Pharmacia, New Jersey, USA). Amplification of the UTRs was performed from poly-T primed-cDNA using specific primers: LbSL (5'CGCTATATAAGTATCAGTTTC3') and Lb181 (5'TGCAACCCGATCATGACCAAG 3') for the 5' UTR, and Lb1824 (5'GATCATGACCAAGATGTACCAGAG 3') and Poly T *Eco*RI (5'CGGAATTCTTTTTTTTTTTTTTTTTTT 3') for the 3' UTR-I (see Additional file 1). Briefly, 20 μL reactions containing 2 μL of cDNA, 1X reaction buffer (10 mM Tris-HCl pH 9.0, 50 mM KCl, 0.1% Triton X-100), 1.5 mM MgCl_2_, 0.7 mM of dNTP mix, 0.2 μM of each primer, 2 M betain, and 0.06 units per μL of expand high fidelity enzyme (Roche, Branford, USA) were prepared. For 3' UTR-II amplification, 1 mM MgCl_2 _and 0.5 μM of each primer were used. An MJ Research PTC-100 DNA thermocycler was used for the reaction with the following amplification profile: 95°C/5 min (initial denaturation), 35 cycles at 92°C/0.5 min, 58°C/0.5 min and 72°C/1 min, with a final incubation at 72°C for 10 min. All the amplified fragments were resolved in agarose gels and visualized under UV exposure after ethidium bromide staining. RT-PCR products were excised from gels, purified using GFX Gel Band Purification kit (Amersham Biosciences, GE Healthcare, Chalfont St. Giles, Buckinghamshire, England) and cloned in the pGEM^®^-T Easy plasmid (Promega, Madison, WI, USA); pCR^®^II (Invitrogen, California, USA) or pCR2.1 (Invitrogen, California, USA) plasmids. The following clones were obtained: pLbHSP70-5B containing the 5' UTR, pTLb3HSP70-D for the 3' UTR-I, pLb3H70-11B for the 3' UTR-II, and pLbHSP70-IR-E for the intercoding region. The sequences were determined using the Big Dye Terminators v3.1 kit (Applied Biosystem, California, USA) by automatic sequencing at the Servicio de Genómica (Parque Científico de Madrid, Universidad Autónoma de Madrid). In order to deduce the *HSP70 *mRNA UTRs from other *Leishmania *species LALIGN (http://www.ch.embnet.org/software/LALIGN_form.html) analyzes were performed. A ClustalW analysis (http://www.ebi.ac.uk/Tools/clustalw2/index.html) for multiple alignments of sequences, were carried out to determine the similarity among them.

## Results and discussion

### The *L. braziliensis HSP70 *locus contains two types of *HSP70 *genes

Gene structure and expression of the HSP70 protein have been well characterized in *L. infantum *[7,15,16,27] and other trypanosomatids of medical importance as *L. major *[28], *Trypanosoma brucei *[29,30], and *Trypanosoma cruzi *[31-36]; nonetheless, little is known about their genes in *L. braziliensis*. A previous study showed the presence of the *HSP70*-*I *genes in this parasite; however, it was not determined their copy number. These authors also reported that using the 3' UTR-II region from *L. infantum *as probe, it was not possible to detect the presence of *HSP70-II *genes in *L. braziliensis *[16].

**Figure 1:**
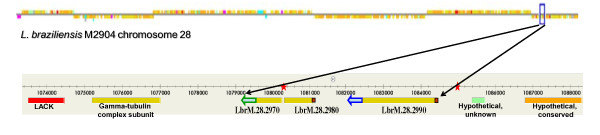
**Location of *HSP70 *genes in the *L. braziliensis *genome**. The upper black arrows demarcate the *HSP70 *locus. Red boxes indicate the 5' UTR, blue arrow the 3 'UTR-I, green arrow the 3 'UTR-II and red stars the gaps in the sequence. Current information on the *L. braziliensis HSP70 *locus at GeneDB database can be accessed through the link: http://www.genedb.org/gene/LbrM.28.2990:mRNA?actionName=%2FQuickSearchQuery#LbrM.28.2990:mRNA

In order to determine the genomic organization of *HSP70 *genes in *L. braziliensis*, we searched in the *L. braziliensis *genome database at GeneDB for entries containing *HSP70 *sequences. One complete *HSP70 *gene (GeneDB ID: LbrM28_v2.2990) and two incomplete sequences, LbrM28_v2.2980 (bearing a 5'-fragment of the gene encoding the N-terminal region of HSP70) and LbrM28_v2.2970 (encoding the C-terminal protein region) were found (Figure 1). Between both fragments of *HSP70 *genes, a sequence gap of undetermined length is annotated in the database. Even though LbrM28_v2.2980 and LbrM28_v2.2970 might be part of the same gene, the sequenced regions do not overlap. All three sequences are tandemly organized on the minus strand of chromosome 28. No other entries for *HSP70 *genes were found in the *L. braziliensis *database. This genomic organization of the *HSP70 *genes in *L. braziliensis *resembled that found in other *Leishmania *species of the subgenus *Leishmania*, i.e. a locus containing tandemly repeated *HSP70 *genes [16]. However, tandem gene arrays are among the most challenging to resolve correctly using current genome sequencing technology, since repetitive sequence reads tend to collapse into a single contig when no variation exists to distinguish them. Thus, in order to determine the copy number of *HSP70 *genes composing the locus, and possible sequence divergence existing between the different genes, we designed specific oligonucleotides to cloning the complete intercoding regions (UTRs+intergenic regions), from genomic DNA, and also the 5'- and 3'-UTRs by RT-PCR from poly-A^+ ^RNA (see Methods section for further details). A single amplification fragment for the intercoding region was obtained and, after cloning, its sequence was found to be conserved (99.8% of sequence identity) with the sequence located between genes LbrM28_v2.2990 and LbrM28_v2.2980. These findings suggest a high conservation of intercoding regions in the *HSP70 *locus; however, a more accurate determination of degree of conservation would require the sequencing of several additional clones.

**Figure 2:**
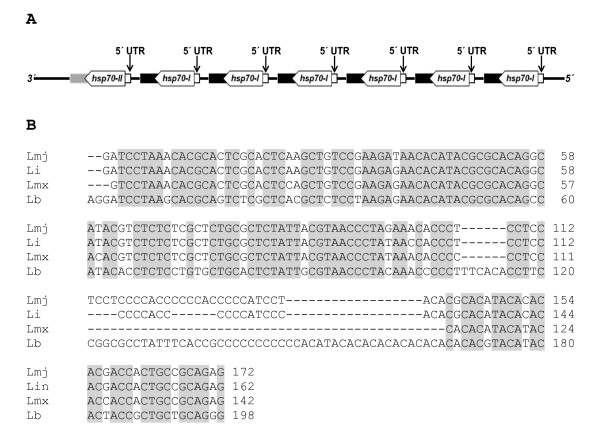
**Position and sequence of the 5´ UTR of HSP70 cluster from L. braziliensis. (A).** Graphical representation of the 5' UTR position in the *HSP70 *cluster of *L. braziliensis*. **(B)**. Multiple sequence alignment among *HSP70 *5' UTR from *L. braziliensis *(Lb: JF449363), *L. major *(Lmj: LmjF28.2770)*, L. infantum *(Li: LinJ28_V3.2960) and *L. mexicana *(Lmx: LmxM28.2770). Conserved sequences are shaded in gray.

On the other hand, using RT-PCR and specific oligonucleotides, it was possible to determine the 5' UTR sequence, which is 198 bp in length (Figure 2). This sequence has been deposited in the GenBank database under the accession number JF449363. In spite of the high sequence identity, among the four sequences analyzed, two microsatellite length polymorphisms in the 5'-UTR were observed (see Additional file 2), suggesting the existence of allelic polymorphisms in the *L. braziliensis HSP70 *locus. In addition, the alignment of the 5' UTR with the genomic sequences allowed us to deduce that the AG splicing acceptor is located 194 and 199 nucleotides upstream of the start codon of LbrM28_v2.2990 and LbrM28_v2.2980 genes, respectively. Also, in both entries, 28 nucleotides upstream of the splicing acceptor site, an identical polypyrimidine tract of 22 nucleotides in length (5' CTCCCCTTTCTCT CTCTGCCCC 3') with a U/C ratio of 0.62 is observed. It is likely that this polypyrimidine tract is relevant for the *trans*-splicing process. Furthermore, in the coding regions contained in clones pLbHSP70-IR-E and pLbHSP70-5B, two transitions of guanine by adenine were found. The first one causes a change of cysteine by tyrosine, and the other produces a change of glutamic acid by lysine in the encoded polypeptides (Additional file 2). This variability, together with the microheterogenicity detected in the 5' UTR, supports the existence of at least four *HSP70 *genes in the diploid genome of *L. braziliensis*; but this resulted to be an underestimation as we demonstrate below.

**Figure 3:**
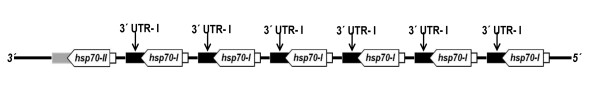
**Graphical representation of the 3' UTR-I position in the *HSP70 *cluster of *L. braziliensis***.

**Figure 4:**
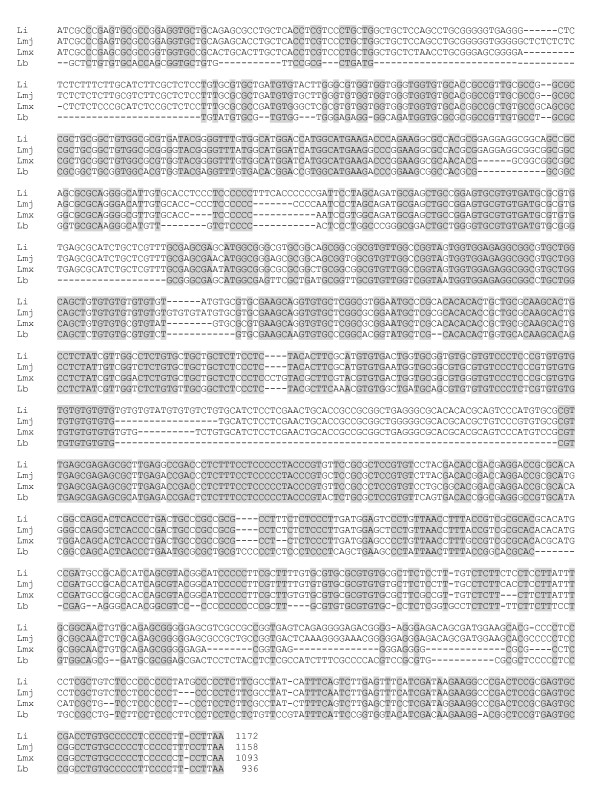
**Multiple sequence alignment among *HSP70 *3' UTR-I from *L. braziliensis *(Lb: JF449364), *L. major *(Lmj: LmjF28.2780)*, L. infantum *(Li: LinJ28_V3.2960) and *L. mexicana *(Lmx)**. Conserved sequences are shaded in gray. *L. major *3' UTR-I sequence was deduced by comparison with *L. infantum *and *L. braziliensis *3' UTR-I sequences. Additional file 3, shows the sequence information, currently available in the GeneDB database, that was used for assembling of the putative *L. mexicana *3' UTR-I sequence.

Regarding the 3' UTR of *HSP70 *genes, two types of sequences were cloned and sequenced (GenBank JF449364 and JF449365), showing a high sequence divergence each other. After sequence comparison, they could be assigned to the two types of 3' UTR (I and II) described in *HSP70 *genes of other *Leishmania *species (Figures 3 and 4). Thus, we determined that the 3' UTR type I is 936 nucleotides long, and would correspond to the LbrM28_v2.2990 entry (and possibly other genes of the *HSP70 *gene cluster, see below), and the 3' UTR type II is 932 nucleotides long and would be associated with LbrM28_v2.2970 entry. The 3' UTR-II was found 100% identical with the genomic sequence located downstream of LbrM28_v2.2970, whereas the 3' UTR-I had 99.1% of sequence identity with the sequence located downstream of LbrM28_v2.2990 entry, suggesting that the cloned 3' UTR-I might correspond to other *HSP70 *gene in the cluster (see below). According to the 3' UTR-I sequence, the polyadenylation in the LbrM28_v2.2990 transcript would occur after two adenines, and 187 bp upstream of the putative polypyrimidine tract previously mentioned. Regarding the LbrM28_v2.2970 gene, the polyadenylation would take place, after an A-rich region of 11 residues, and 154 bp upstream of a C-rich polypyrimidine tract of 14 nucleotides in length and U/C ratio of 0.08 (5'CCCCCCCCCTCCCC 3'). It is a common feature that the presence of adenosine residue precedes the polyadenylation sites of a large number of trypanosome mRNAs [37]. It is believed that poly(A) polymerases prefer an initial adenosine residue for attachment of the poly(A) tail, and the selection of the polyadenylation site would be strengthened by the presence of adenosine residues [38].

After determining the extent of the UTRs, it was possible to define the intergenic region (IR) within the *L. braziliensis HSP70 *locus. The IR, not included in the mature mRNA, is defined as the sequence beginning downstream of the polyadenylation site and ending immediately upstream of the spliced leader acceptor site of a downstream gene. Thus, the IR between LbrM28.2990 and LbrM28.2980 genes was found to be 237 nucleotides in length; this region was almost identical (99.6%) to that cloned for this work (pLbHSP70-IR-E clone, GenBank accession number JF449366), suggesting a high degree of conservation of this region along the *HSP70 *cluster.

### Comparison of *L. braziliensis HSP70 *UTRs with their homologues in other *Leishmania *species

The characterization of UTRs for *L. braziliensis HSP70 *genes (Figures 2, 3 and 4) has evidenced the existence of a remarkable conservation of the *HSP70 *locus along the genus *Leishmania*, extending previous studies [16] to a species of the *Viannia *subgenus. The 5' UTR cloned in this work was found to be highly conserved with the equivalent regions of the LbrM28_v2.2990 and LbrM28_v2.2980 genes (98% and 99.5%, respectively). The comparison with the *HSP70 *5' UTR of other *Leishmania *species showed also a remarkable sequence identity (77-81%). Noticeably, this region was well conserved among all *Leishmania *species analyzed except for two exclusive sequences of *L. braziliensis *(Figure 2).

Comparison of the 3' UTR-I from *L. braziliensis *with those from other *Leishmania *species revealed identities between 71-73%. Furthermore, stretches of identical nucleotides are present in the 3' UTR-I in all the species analyzed (Figure 3 and 4). Again, it was found that *L. braziliensis *sequence is the most divergent among the analyzed sequences. Thus, the *L. braziliensis *3' UTR-I lacks several regions common to the other *Leishmania *species; in particular, there are two important sequence gaps, of 60 and 77 nts, located at the beginning and the middle of the region, respectively (Figure 3 and 4).

**Figure 5:**
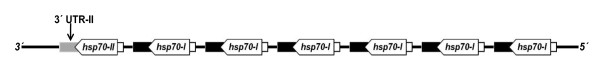
**Graphical representation of the 3' UTR-II position in the *HSP70 *cluster of *L. braziliensis***.

**Figure 6:**
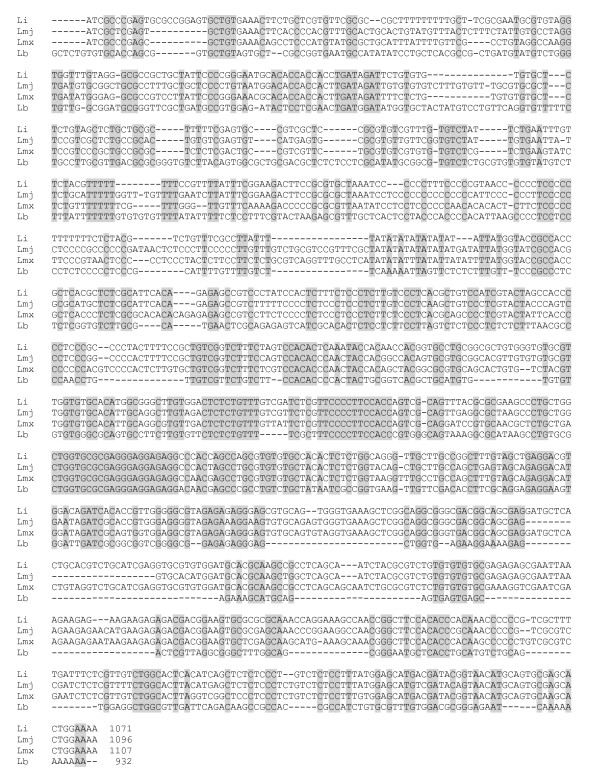
**Multiple sequence alignment among *HSP70 *3'UTR-II from *L. braziliensis *(Lb: JF449365), *L. major *(Lmj: LmjF28.2770)*, L. infantum *(Li: LinJ28_V3.3000) and *L. mexicana *(Lmx: LmxM.28.2770)**. Conserved sequences are shaded in gray. *L. major *and *L. mexicana *sequences were deduced by comparison with *L. infantum *and *L. braziliensis *3' UTR-II sequences.

In contrast to the 3' UTR-I, the 3' UTR-II from *L. braziliensis *showed to be more divergent, having only a 55-57% of identity with the other species analyzed. Indeed, some sequences at the first half of the region were exclusive for *L. braziliensis *(Figure 5 and 6). Conversely, it was found that *L. braziliensis *sequence lacks several regions common to the other *Leishmania *species, especially in the second half of the region (Figure 5 and 6). Nevertheless, the presence of several short stretches of identical sequences among all the species analyzed was also found (Figure 5 and 6), suggesting a common evolutionarily origin.

### Expression analysis of *HSP70 *genes in *L. braziliensis*

**Figure 7:**
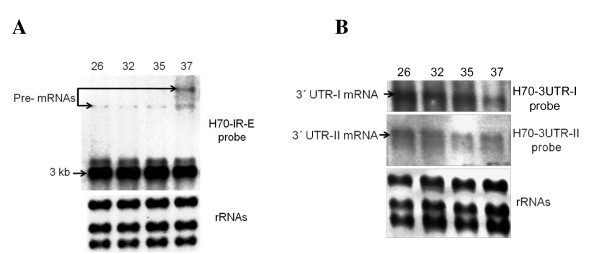
**Identification of *HSP70-I *and *HSP70-II *transcripts of *L. braziliensis***. Four micrograms of total RNA isolated from promastigotes incubated for 2 h at the indicated temperatures were separated on denaturant 1.5% agarose/MOPS/formaldehyde gel, blotted and hybridized with the H70-IR-E **(A)**, 3' UTR-I, and 3' UTR-II **(B)** probes. As load control, the agarose gels were stained with ethidium bromide (rRNA panels).

The identification of two divergent 3' UTR sequences for *HSP70 *genes in *L. braziliensis *and their comparison with the sequences in the GeneDB database allowed us to define the existence of two types of *HSP70 *genes, a question that remained to be solved [16]. Northern blot assays using different probes were performed in order to elucidate the expression of these genes in *L. braziliensis*. Using the IR-HSP70 probe, containing the complete 3'-UTR-I together with 5'-UTR and short fragment of the coding region, two hybridization RNA species of very similar size were observed (Figure 7). In addition, a slight hybridizing fragment of about 5.6 kb was observed in all lanes, but its signal became stronger when parasites were incubated at 37°C; accumulation of additional high molecular RNA species was also observed with this treatment (Figure 7A). It is likely that those fragments are representing RNA precursors (pre-mRNA), whose processing is disturbed by heat shock. The use of probes specific for 3' UTR-I and UTR-II allowed us to show the existence of both types of transcripts: the *HSP70-II *mRNA, which corresponds to the upper RNA in figure 7A, and the *HSP70-I *mRNA, which corresponds to the lower one. Of special interest, both types of transcripts did not show accumulation under heat shock treatment (Figure 7).

### Copy number and chromosomal location of *HSP70 *genes in *L. braziliensis*

**Figure 8:**
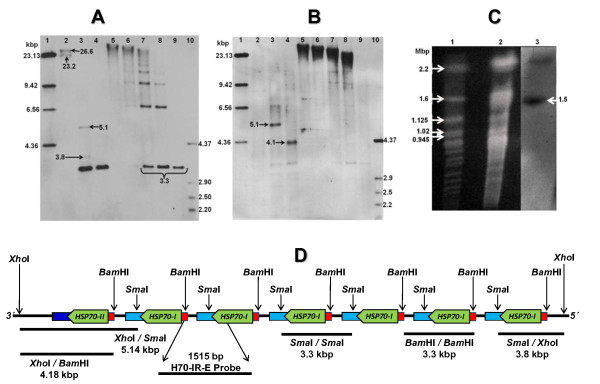
**Genomic organization of *HSP70 *genes from *L. braziliensis***. DNA from promastigotes was totally or partially digested with restriction enzymes and the resulting fragments were separated on a 0.8% agarose gel, transferred to nylon membrane and hybridized with the H70-IR-E probe **(A)**. The same blot was stripped and rehybridized with the *HSP70*-3' UTR-II probe **(B)**. Lanes: 1, 185 ng of λ DNA *Hind*III; 2, 300 ng of *Xho*I-digested DNA; 3, 1 μg of *Xho*I+*Sma*I-digested DNA; 4, 1 μg of *Xho*I+*Bam*HI-digested DNA; 5 to 9, DNA digested with *Bam*HI for 2 min (lane 5), 5 min (lane 6), 15 min (lane 7), 30 min (lane 8), and 3 hours (lane 9); lane 10, 140 ng of Φ29 DNA+*Hin*dIII. Molecular weight markers (lane 1 and 10) were labeled with digoxigenin and used as a probe in the hybridizations. **(C)** Pulsed field gel electrophoresis showing ethidium bromide staining of *S. cerevisiae *(lane 1), and *L. braziliensis *chromosomes (lane 2). Panel 3 shows the hybridization of the H70-IR-E probe to the *L. braziliensis *chromosomes. **(D)** Graphical representation of the deduced physical map for *HSP70 *locus in *L. braziliensis*. Small red boxes represent the 5' UTR; light blue boxes the 3' UTR-I, dark blue one the 3' UTR-II, and green boxes the ORFs.

In order to estimate the number of *HSP70 *genes in the *L. braziliensis *locus, a Southern blot analysis was carried out (Figure 8). *L. braziliensis *total DNA was either totally or partially digested with selected restriction enzymes, and, after electrophoretic separation and transferring, hybridized with specific probes. A prominent 3.3-kb fragment was observed after digesting DNAs with either *Sma*I- or *Bam*HI and hybridizing with the intercoding probe (H70-IR-E; Figure 8A), indicating that indeed the *HSP70 *locus in *L. braziliensis *should consist of tandemly repeated genes with a repetition unit of 3.3-kb. Additional fragments, showing lower hybridizations signals, were interpreted as representing the boundaries of the locus (Figure 8A, lane 3, and 8B, lanes 3, and 4). The tandem organization of the locus was demonstrated by *Bam*HI partial digestion of *L. braziliensis *DNA; a typical ladder was observed (Figure 8A, lanes 5 to 8).

Hybridization of the membrane with the 3' UTR-II probe showed a sole hybridization fragment (5.1-kb in *Xho*I+*Sma*I digested DNA, and 4.1-kb in *Xho*I+*Bam*HI digested DNA) (Figure 8B, lanes 3 and 4, respectively), in agreement with the existence of a unique *HSP70*-II gene, which was located at the 3' end of the locus (Figure 8D). According to the number of fragments observed in the lanes containing *Bam*HI partially digested DNA (Figure 8A, lanes 5 to 8), and taking into account the size of the hybridizing *Xho*I-fragments which should contain the complete locus (Figure 8A, lane 2), it was estimated the presence of around seven *HSP70 *genes in the *L. braziliensis HSP70 *locus, as is shown in Figure 8D which summarizes the genomic organization of the *L. braziliensis HSP70 *locus.

The presence of two hybridization fragments, larger than 20 kb, in the lane containing *Xho*I-digested DNA (Figure 8A, lane 2) has no direct explanation. Two hypotheses may be invoked to explain this unexpected finding. On the one hand, it can be postulated the existence of two identical *HSP70 *tandems that, furthermore, should be found in the same chromosome. Thus, PFGE separation and hybridization assays showed that *HSP70 *genes are located in a chromosome of approximately 1.5 Mb (Figure 8C), which would correspond to the chromosome 28, according to the location of the *HSP70 *locus in the GeneDB database. The other hypothesis is the existence of allelic polymorphisms either within the locus (affecting the number of *HSP70 *gene copies) or in its boundaries (affecting one of the *Xho*I restriction sites). Although the size and hybridization intensity of these *Xho*I restriction fragments supports the second alternative, and the resolution of the PFGE assay do not permit distinguish between the two size different sister chromosomes, further work is needed to clarify this finding.

## Conclusions

The present work was intended to establish the genomic organization of *HSP70 *genes in *L. braziliensis. *Firstly, by RT-PCR and cloning, we identified two types of 3' UTR sequences, demonstrating that also in *L. braziliensis *two types of *HSP70 *genes exist, a feature shared with other *Leishmania *species. In addition, our analyses support the existence of at least seven *HSP70 *genes arranged in a head-to-tail manner. In summary, the *HSP70 *locus in *L. braziliensis*, like in *Leishmania *subgenus species, is composed of the two types of genes (*HSP70-I *and *HSP70-II*), the number and the relative position of these genes being very similar in the *Leishmania *genus. This finding is of especial value taking into account that *L. (V.) braziliensis *complex is considered as the earliest divergent species of the genus *Leishmania *[39]. The strict conservation of the *HSP70 *gene array in all *Leishmania *species analyzed suggests that this type of arrangement must have an important functional role. Indeed, as recently reported, the *HSP70-II *gene in *L. infantum *is extremely relevant for virulence and intracellular survival of the parasite [40].

Additionally, we have experimentally mapped the 5' and 3' UTR of both types of *HSP70 *genes of *L. braziliensis*. After comparing with the genomic sequences, the position of processing signals, such as the *trans*-splicing and polyadenylation sites as well as the C-rich polypyrimidine tracts, were determined. The distance between these elements is in agreement with previously reported range of distances [41].

Former studies in *L. braziliensis *reported that after probing the mRNA blot with the *HSP70 *coding region, two hybridization RNA species very close in size were observed, corresponding the lower molecular weight species to that hybridizing with a 3' UTR-I probe [16]. Our Northern blot analysis supported these findings and revealed that the larger RNA corresponds to the *HSP70-II *transcript, confirming the existence of the *HSP70-II *genes in this parasite. Although, the size difference between these transcripts could not be explained by the sole difference in size of both 3' UTRs, it is likely that the differences are due to different length of the poly(A) tail [42,43]. Noticeably, it is considered that unstable mRNAs carry shorter poly (A) tails [44,45]. Northern blot assays showed that the steady-state levels of both transcripts are not affected by the temperature of incubation. Consequently, in contrast to the species of the *Leishmania *subgenus, *L. braziliensis HSP70-I *transcripts are not stabilized upon heat shock.

## Abbreviations

CL: cutaneous leishmaniasis; MCL: muco-cutaneos leishmaniasis; HSP: heat shock protein; HSP70: 70 kDa heat shock protein; IR: Intergenic region; PFGE: pulsed field gel electrophoresis; PPT: thermosensitive polypyrimidine-rich element; UTR: untranslated region; VL: visceral leishmaniasis; LACK: activated protein kinase c receptor (guanine nucleotide-binding protein beta subunit- like protein).

## Competing interests

The authors declare that they have no competing interests.

## Authors' contributions

CAR, JMR, and CJP conceived and designed the experiments. CAR performed the experiments. CAR, JMR, and CJP analyzed the data. CAR, JMR, and CJP wrote the paper. All authors revised and approved the final version of the manuscript.

## Supplementary Material

Additional file 1 **Strategy for cloning the intercoding and UTR regions of *L. braziliensis HSP70 *genes**. **(A) **Location of the primers according to the *L. braziliensis *genome database. (**B**) Amplification of the intercoding region using the Lb1817F/Lb181R primers (pLbHSP70-IR-E clone). (**C**) Total RNA was extracted and cDNA synthesized with polyT-*Eco*RI primer. The cDNA was used as template to amplify the 5' UTR, using the LbSLF and Lb181R primers (pLbHSP70-5B clone), or 3' UTRs, using the Lb1824F and polyT-*Eco*RI primers. pTLb3HSP70-D, and pLb3H70-11B clones correspond to 3' UTR-I, and 3' UTR-II, respectively. Click here for file

Additional file 2 **CLUSTAL 2.1 multiple sequence alignment between the sequence of pLbHSP70-5B clone and the equivalent sequences present in pLbHSP70-IR-E clone, and genes LbrM28_V2.2990 and LbrM28_V2.2980**. Shaded in grey is the start codon; LbrM28_V2.2980 gene has in the 5' UTR an additional cytosine in position 138 (in bold); the 5' UTR from LbrM28_V2.2990 gene contains four nucleotide gaps (underlined in the other sequences). In position 248 (shaded in green) of pLbHSP70-IR-E sequence, within the coding sequence, there is a G to A transition that generates a change of cysteine (C) by tyrosine (Y) in the protein. In position 340 (shaded in fuchsia) of pLbHSP70-5B sequence, there is other transition of a guanine for an adenine that generates a change of Glutamic acid (E) by Lysine (K). Click here for file

Additional file 3 **Location of *HSP70 *genes in the *L. mexicana *genome**. The upper black arrows demarcate the *HSP70 *locus. Red boxes indicate the 5' UTR, blue arrows the 3' UTR-I, green arrow the 3' UTR-II, and red stars the gaps in the sequence. *L. mexicana *3' UTR-I sequence was assembled from three fragments (GeneDB positions: 1'048.970 - 1'049.137; 1'047.042 - 1'047.656, and 1'045.959 - 1'046.951) deduced by comparison of the *L. mexicana *genome with the GenBank entry L14605.1, a sequence containing the 3'UTR-I from *L. mexicana amazonensis*. *L. mexicana *3' UTR-II sequence was deduced by comparison with *L. infantum *and *L. braziliensis *3' UTR-II sequences. Click here for file

## References

[B1] Murray HW Berman JD Davies CR Saravia NG Advances in leishmaniasis Lancet 2005 366 9496 1561 1577 10.1016/S0140-6736(05)67629-5 16257344

[B2] Control of the leishmaniases http://whqlibdoc.who.int/trs/WHO_TRS_949_eng.pdf

[B3] Oddone R Schweynoch C Schonian G de Sousa Cdos S Cupolillo E Espinosa D Arevalo J Noyes H Mauricio I Kuhls K Development of a multilocus microsatellite typing approach for discriminating strains of *Leishmania (Viannia)* species J Clin Microbiol 2009 47 9 2818 2825 10.1128/JCM.00645-09 19587302 PMC2738093

[B4] Dantas-Torres F Canine leishmaniosis in South America Parasit Vectors 2009 2 Suppl 1 S1 10.1186/1756-3305-2-S1-S1 19426440 PMC2679393

[B5] Shapira M McEwen JG Jaffe CL Temperature effects on molecular processes which lead to stage differentiation in *Leishmania* EMBO J 1988 7 9 2895 2901 3181145 10.1002/j.1460-2075.1988.tb03147.x PMC457084

[B6] Maresca B Kobayashi GS Hsp70 in parasites: as an inducible protective protein and as an antigen Experientia 1994 50 11-12 1067 1074 10.1007/BF01923463 7988666

[B7] Quijada L Soto M Alonso C Requena JM Analysis of post-transcriptional regulation operating on transcription products of the tandemly linked *Leishmania infantum* hsp70 genes J Biol Chem 1997 272 7 4493 4499 10.1074/jbc.272.7.4493 9020174

[B8] Krobitsch S Clos J A novel role for 100 kD heat shock proteins in the parasite Leishmania donovani Cell Stress Chaperones 1999 4 3 191 198 10.1379/1466-1268(1999)004<0191:ANRFKH>2.3.CO;2 10547068 PMC312933

[B9] Zilka A Garlapati S Dahan E Yaolsky V Shapira M Developmental regulation of heat shock protein 83 in *Leishmania*. 3' processing and mRNA stability control transcript abundance, and translation id directed by a determinant in the 3'-untranslated region J Biol Chem 2001 276 51 47922 47929 11598129 10.1074/jbc.M108271200

[B10] Bente M Harder S Wiesgigl M Heukeshoven J Gelhaus C Krause E Clos J Bruchhaus I Developmentally induced changes of the proteome in the protozoan parasite *Leishmania donovani* Proteomics 2003 3 9 1811 1829 10.1002/pmic.200300462 12973740

[B11] Hartl FU Molecular chaperones in cellular protein folding Nature 1996 381 6583 571 579 10.1038/381571a0 8637592

[B12] Bukau B Horwich AL The Hsp70 and Hsp60 chaperone machines Cell 1998 92 3 351 366 10.1016/S0092-8674(00)80928-9 9476895

[B13] Mayer MP Bukau B Hsp70 chaperones: cellular functions and molecular mechanism Cell Mol Life Sci 2005 62 6 670 684 10.1007/s00018-004-4464-6 15770419 PMC2773841

[B14] Folgueira C Requena JM A postgenomic view of the heat shock proteins in kinetoplastids FEMS Microbiol Rev 2007 31 4 359 377 10.1111/j.1574-6976.2007.00069.x 17459115

[B15] Folgueira C Quijada L Soto M Abanades DR Alonso C Requena JM The translational efficiencies of the two *Leishmania infantum* HSP70 mRNAs, differing in their 3'-untranslated regions, are affected by shifts in the temperature of growth through different mechanisms J Biol Chem 2005 280 42 35172 35183 10.1074/jbc.M505559200 16105831

[B16] Folgueira C Canavate C Chicharro C Requena JM Genomic organization and expression of the HSP70 locus in New and Old World *Leishmania* species Parasitology 2007 134 Pt 3 369 377 17054823 10.1017/S0031182006001570

[B17] Requena JM Lights and shadows on gene organization and regulation of gene expression in *Leishmania* Front Biosci 2011 17 2069 2085 21622163 10.2741/3840

[B18] Charest H Zhang WW Matlashewski G The developmental expression of *Leishmania donovani* A2 amastigote-specific genes is post-transcriptionally mediated and involves elements located in the 3'-untranslated region J Biol Chem 1996 271 29 17081 17090 10.1074/jbc.271.29.17081 8663340

[B19] Mishra KK Holzer TR Moore LL LeBowitz JH A negative regulatory element controls mRNA abundance of the *Leishmania mexicana* Paraflagellar rod gene PFR2 Eukaryot Cell 2003 2 5 1009 1017 10.1128/EC.2.5.1009-1017.2003 14555483 PMC219351

[B20] Larreta R Soto M Quijada L Folgueira C Abanades DR Alonso C Requena JM The expression of HSP83 genes in *Leishmania infantum* is affected by temperature and by stage-differentiation and is regulated at the levels of mRNA stability and translation BMC Mol Biol 2004 5 3 10.1186/1471-2199-5-3 15176985 PMC436058

[B21] Purdy JE Donelson JE Wilson ME *Leishmania chagasi*: the alpha-tubulin intercoding region results in constant levels of mRNA abundance despite protein synthesis inhibition and growth state Exp Parasitol 2005 110 2 102 107 10.1016/j.exppara.2005.02.008 15888291

[B22] Zick A Onn I Bezalel R Margalit H Shlomai J Assigning functions to genes: identification of S-phase expressed genes in *Leishmania major* based on post-transcriptional control elements Nucleic Acids Res 2005 33 13 4235 4242 10.1093/nar/gki742 16052032 PMC1181863

[B23] Holzer TR Mishra KK LeBowitz JH Forney JD Coordinate regulation of a family of promastigote-enriched mRNAs by the 3'UTR PRE element in *Leishmania mexicana* Mol Biochem Parasitol 2008 157 1 54 64 10.1016/j.molbiopara.2007.10.001 18023890 PMC2692640

[B24] David M Gabdank I Ben-David M Zilka A Orr I Barash D Shapira M Preferential translation of Hsp83 in *Leishmania* requires a thermosensitive polypyrimidine-rich element in the 3' UTR and involves scanning of the 5' UTR RNA 2010 16 2 364 374 10.1261/rna.1874710 20040590 PMC2811665

[B25] Peacock CS Seeger K Harris D Murphy L Ruiz JC Quail MA Peters N Adlem E Tivey A Aslett M Comparative genomic analysis of three *Leishmania* species that cause diverse human disease Nat Genet 2007 39 7 839 847 10.1038/ng2053 17572675 PMC2592530

[B26] Sambrook JRD Molecular Cloning: A Laboratory Manual 2001 2 New York

[B27] Quijada L Soto M Alonso C Requena JM Identification of a putative regulatory element in the 3'-untranslated region that controls expression of HSP70 in *Leishmania infantum* Mol Biochem Parasitol 2000 110 1 79 91 10.1016/S0166-6851(00)00258-9 10989147

[B28] Lee MG Atkinson BL Giannini SH Van der Ploeg LH Structure and expression of the hsp 70 gene family of *Leishmania major* Nucleic Acids Res 1988 16 20 9567 9585 10.1093/nar/16.20.9567 3186441 PMC338764

[B29] Hausler T Clayton C Post-transcriptional control of hsp70 mRNA in *Trypanosoma brucei* Mol Biochem Parasitol 1996 76 1-2 57 71 10.1016/0166-6851(95)02538-3 8919995

[B30] Lee MG The 3' untranslated region of the hsp 70 genes maintains the level of steady state mRNA in *Trypanosoma brucei* upon heat shock Nucleic Acids Res 1998 26 17 4025 4033 10.1093/nar/26.17.4025 9705515 PMC147808

[B31] Engman DM Sias SR Gabe JD Donelson JE Dragon EA Comparison of HSP70 genes from two strains of *Trypanosoma cruzi* Mol Biochem Parasitol 1989 37 2 285 287 10.1016/0166-6851(89)90161-8 2691890

[B32] Requena JM Lopez MC Jimenez-Ruiz A de la Torre JC Alonso C A head-to-tail tandem organization of hsp70 genes in *Trypanosoma cruzi* Nucleic Acids Res 1988 16 4 1393 1406 10.1093/nar/16.4.1393 2831499 PMC336323

[B33] Requena JM Lopez MC Jimenez-Ruiz A Morales G Alonso C Complete nucleotide sequence of the hsp70 gene of *T. cruzi* Nucleic Acids Res 1989 17 2 797 10.1093/nar/17.2.797 2644623 PMC331622

[B34] Requena JM Jimenez-Ruiz A Soto M Assiego R Santaren JF Lopez MC Patarroyo ME Alonso C Regulation of hsp70 expression in *Trypanosoma cruzi *by temperature and growth phase Mol Biochem Parasitol 1992 53 1-2 201 211 10.1016/0166-6851(92)90022-C 1501640

[B35] de Carvalho EF de Castro FT Rondinelli E Soares CM Carvalho JF HSP 70 gene expression in *Trypanosoma cruzi* is regulated at different levels J Cell Physiol 1990 143 3 439 444 10.1002/jcp.1041430306 2193034

[B36] Rodrigues DC Silva R Rondinelli E Urmenyi TP *Trypanosoma cruzi*: modulation of HSP70 mRNA stability by untranslated regions during heat shock Exp Parasitol 2010 126 2 245 253 10.1016/j.exppara.2010.05.009 20493845

[B37] Matthews KR Tschudi C Ullu E A common pyrimidine-rich motif governs trans-splicing and polyadenylation of tubulin polycistronic pre-mRNA in trypanosomes Genes Dev 1994 8 4 491 501 10.1101/gad.8.4.491 7907303

[B38] Stiles JK Hicock PI Shah PH Meade JC Genomic organization, transcription, splicing and gene regulation in *Leishmania* Ann Trop Med Parasitol 1999 93 8 781 807 10.1080/00034989957781 10715672

[B39] Momen H Cupolillo E Speculations on the origin and evolution of the genus *Leishmania* Mem Inst Oswaldo Cruz 2000 95 4 583 588 10.1590/S0074-02762000000400023 10904419

[B40] Carrion J Folgueira C Soto M Fresno M Requena JM *Leishmania infantum* HSP70-II null mutant as candidate vaccine against leishmaniasis: a preliminary evaluation Parasit Vectors 2011 4 1 150 10.1186/1756-3305-4-150 21794145 PMC3199857

[B41] LeBowitz JH Smith HQ Rusche L Beverley SM Coupling of poly(A) site selection and trans-splicing in *Leishmania* Genes Dev 1993 7 6 996 1007 10.1101/gad.7.6.996 8504937

[B42] Dewannieux M Heidmann T Role of poly(A) tail length in Alu retrotransposition Genomics 2005 86 3 378 381 10.1016/j.ygeno.2005.05.009 15993034

[B43] van Leeuwen HC Liefhebber JM Spaan WJ Repair and polyadenylation of a naturally occurring hepatitis C virus 3' nontranslated region-shorter variant in selectable replicon cell lines J Virol 2006 80 9 4336 4343 10.1128/JVI.80.9.4336-4343.2006 16611892 PMC1472026

[B44] Shapira M Zilka A Garlapati S Dahan E Dahan I Yavesky V Post transcriptional control of gene expression in *Leishmania* Med Microbiol Immunol 2001 190 1-2 23 26 11770103 10.1007/s004300100073

[B45] Schwede A Ellis L Luther J Carrington M Stoecklin G Clayton C A role for Caf1 in mRNA deadenylation and decay in trypanosomes and human cells Nucleic Acids Res 2008 36 10 3374 3388 10.1093/nar/gkn108 18442996 PMC2425496

