# Supplementary figures and images for: Identification of the HSP70-II gene in Leishmania braziliensis HSP70 locus: genomic organization and UTRs characterization
Source: Parasit Vectors. 2011 Aug 26;4:166. doi: 10.1186/1756-3305-4-166 (PMC3185273; doi:10.1186/1756-3305-4-166)

**A**

*L. braziliensis* M2904 chromosome 28

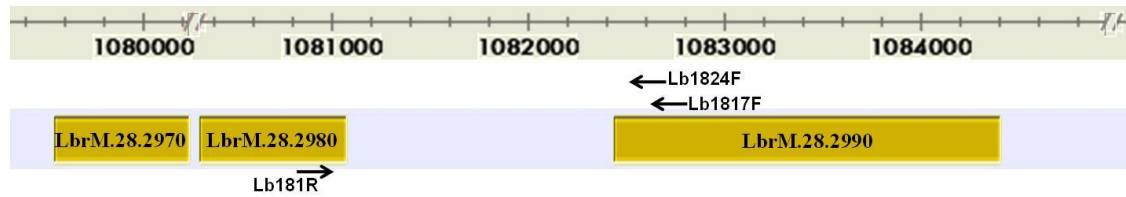

**B**

PCR using genomic DNA as template

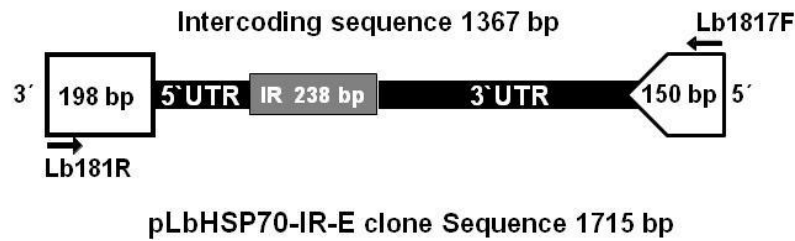

**C**

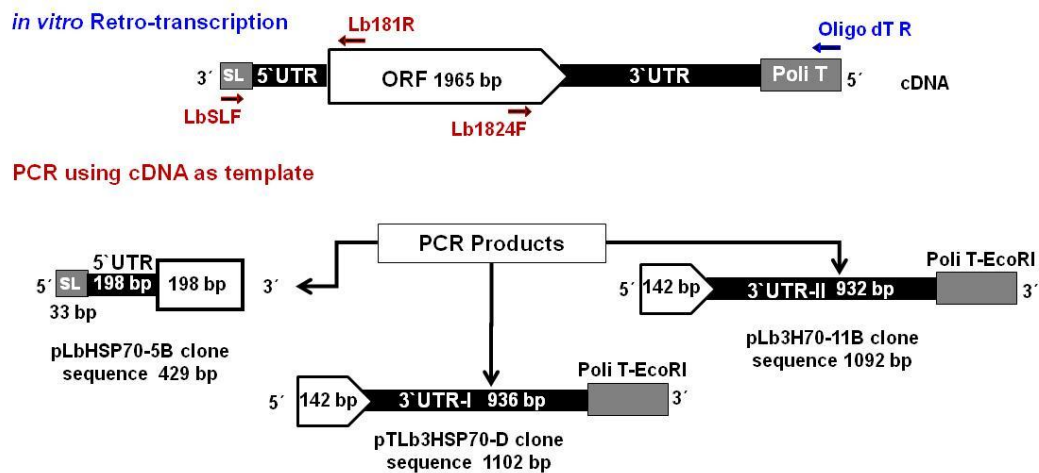

Supplementary material 1.

Supplement: Additional file 1 — Strategy for cloning the intercoding and UTR regions of L. braziliensis HSP70 genes. (A) Location of the primers according to the L. braziliensis genome database. (B) Amplification of the intercoding region using the Lb1817F/Lb181R primers (pLbHSP70-IR-E clone). (C) Total RNA was extracted and cDNA synthesized with polyT-EcoRI primer. The cDNA was used as template to amplify the 5' UTR, using the LbSLF and Lb181R primers (pLbHSP70-5B clone), or 3' UTRs, using the Lb1824F and polyT-EcoRI primers. pTLb3HSP70-D, and pLb3H70-11B clones correspond to 3' UTR-I, and 3' UTR-II, respectively. [file 1756-3305-4-166-S1.PDF]

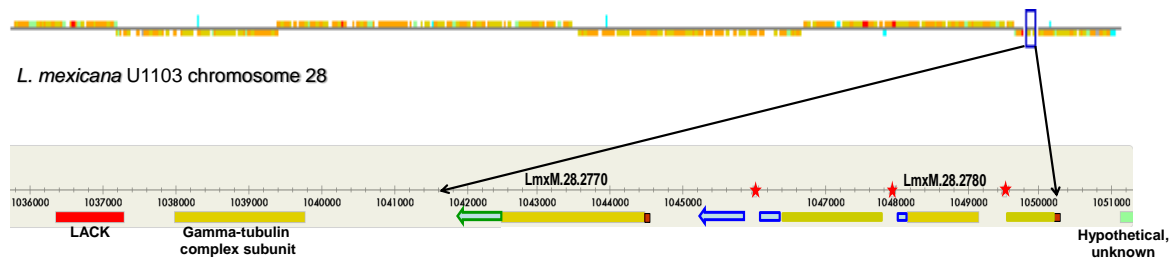

Supplementary Material 3

Supplement: Additional file 3 — Location of HSP70 genes in the L. mexicana genome. The upper black arrows demarcate the HSP70 locus. Red boxes indicate the 5' UTR, blue arrows the 3' UTR-I, green arrow the 3' UTR-II, and red stars the gaps in the sequence. L. mexicana 3' UTR-I sequence was assembled from three fragments (GeneDB positions: 1'048.970 - 1'049.137; 1'047.042 - 1'047.656, and 1'045.959 - 1'046.951) deduced by comparison of the L. mexicana genome with the GenBank entry L14605.1, a sequence containing the 3'UTR-I from L. mexicana amazonensis. L. mexicana 3' UTR-II sequence was deduced by comparison with L. infantum and L. braziliensis 3' UTR-II sequences. [file 1756-3305-4-166-S3.PDF]
