# Supplementary material for: Identification of the HSP70-II gene in Leishmania braziliensis HSP70 locus: genomic organization and UTRs characterization
Source: Parasit Vectors. 2011 Aug 26;4:166. doi: 10.1186/1756-3305-4-166 (PMC3185273; doi:10.1186/1756-3305-4-166)
Supplement: Additional file 2 — CLUSTAL 2.1 multiple sequence alignment between the sequence of pLbHSP70-5B clone and the equivalent sequences present in pLbHSP70-IR-E clone, and genes LbrM28_V2.2990 and LbrM28_V2.2980. Shaded in grey is the start codon; LbrM28_V2.2980 gene has in the 5' UTR an additional cytosine in position 138 (in bold); the 5' UTR from LbrM28_V2.2990 gene contains four nucleotide gaps (underlined in the other sequences). In position 248 (shaded in green) of pLbHSP70-IR-E sequence, within the coding sequence, there is a G to A transition that generates a change of cysteine (C) by tyrosine (Y) in the protein. In position 340 (shaded in fuchsia) of pLbHSP70-5B sequence, there is other transition of a guanine for an adenine that generates a change of Glutamic acid (E) by Lysine (K). [file 1756-3305-4-166-S2.PDF]

```
LbrM28_V2.2980    AGGATCCTAAGCACGCAGTCTCGCTCACGCTCTCCTAAGAGAACACATACGCGCACAGCC  60
pLbHSP70-IR-E     AGGATCCTAAGCACGCAGTCTCGCTCACGCTCTCCTAAGAGAACACATACGCGCACAGCC  60
pLbHSP70-5B       AGGATCCTAAGCACGCAGTCTCGCTCACGCTCTCCTAAGAGAACACATACGCGCACAGCC  60
LbrM28_V2.2990    AGGATCCTAAGCACGCAGTCTCGCTCACGCTCTCCTAAGAGAACACATACGCGCACAGCC  60

LbrM28_V2.2980    ATACACCTCTCCTGTGCTGCACTCTATTGCGTAACCCTACAAACCCCCTTTCACACCTTC 120
pLbHSP70-IR-E     ATACACCTCTCCTGTGCTGCACTCTATTGCGTAACCCTACAAACCCCCTTTCACACCTTC 120
pLbHSP70-5B       ATACACCTCTCCTGTGCTGCACTCTATTGCGTAACCCTACAAACCCCCTTTCACACCTTC 120
LbrM28_V2.2990    ATACACCTCTCCTGTGCTGCACTCTATTGCGTAACCCTACAAACCCCCTTTCACACCTTC 120

LbrM28_V2.2980    CGGCGCCTATTTCACCGCCCCCCCCCCCCACATACACACACACACACACACACGTACATA 180
pLbHSP70-IR-E     CGGCGCCTATTTCACCG-CCCCCCCCCCCACATACACACACACACACACACACGTACATA 179
pLbHSP70-5B       CGGCGCCTATTTCACCG-CCCCCCCCCCCACATACACACACACACACACACACGTACATA 179
LbrM28_V2.2990    CGGCGCCTATTTCACCG-CCCCCCCCCCCACAT----ACACACACACACACACGTACATA 175

LbrM28_V2.2980    CACTACCGCTGCTGCAGGGATGACGTTCGAGGGCGCCATTGGCATCGACCTGGGCACGAC 240
pLbHSP70-IR-E     CACTACCGCTGCTGCAGGGATGACGTTCGAGGGCGCCATTGGCATCGACCTGGGCACGAC 239
pLbHSP70-5B       CACTACCGCTGCTGCAGGGATGACGTTCGAGGGCGCCATTGGCATCGACCTGGGCACGAC 239
LbrM28_V2.2990    CACTACCGCTGCTGCAGGGATGACGTTCGAGGGCGCCATTGGCATCGACCTGGGCACGAC 235

LbrM28_V2.2980    GTACTCGTGCGTGGGCGTGTGGCAGAACGAGCGCGTGGAGATCATCGCGAACGACCAGGG 300
pLbHSP70-IR-E     GTACTCGTACGTGGGCGTGTGGCAGAACGAGCGCGTGGAGATCATCGCGAACGACCAGGG 299
pLbHSP70-5B       GTACTCGTGCGTGGGCGTGTGGCAGAACGAGCGCGTGGAGATCATCGCGAACGACCAGGG 299
LbrM28_V2.2990    GTACTCGTGCGTGGGCGTGTGGCAGAACGAGCGCGTGGAGATCATCGCGAACGACCAGGG 295

LbrM28_V2.2980    TAACCGCACGACGCCGTCGTACGTTGCGTTCACGGACTCGGAGCGCCTGATCGGCGATGC 360
pLbHSP70-IR-E     TAACCGCACGACGCCGTCGTACGTTGCGTTCACGGACTCGGAGCGCCTGATCGGCGATGC 359
pLbHSP70-5B       TAACCGCACGACGCCGTCGTACGTTGCGTTCACGGACTCGAAGCGCCTGATCGGCGATGC 359
LbrM28_V2.2990    TAACCGCACGACGCCGTCGTACGTTGCGTTCACGGACTCGGAGCGCCTGATCGGCGATGC 355

LbrM28_V2.2980    CGCGAAGAACCAGGTGGCGATGAACCCGCACAACACG 397
pLbHSP70-IR-E     CGCGAAGAACCAGGTGGCGATGAACCCGCACAACACG 396
pLbHSP70-5B       CGCGAAGAACCAGGTGGCGATGAACCCGCACAACACG 396
LbrM28_V2.2990    CGCGAAGAACCAGGTGGCGATGAACCCGCACAACACG 392
```

Supplementary material 2.
